# Study of Methanol Extracts from Different Parts of *Peganum harmala* L. Using ^1^H-NMR Plant Metabolomics

**DOI:** 10.1155/2018/6532789

**Published:** 2018-11-18

**Authors:** Yinping Li, Qing He, Shushan Du, Shanshan Guo, Zhufeng Geng, Zhiwei Deng

**Affiliations:** ^1^College of Chemistry, Beijing Normal University, Beijing 100875, China; ^2^College of Chemistry and Chemical Engineering, Xinjiang Normal University, Urumqi, Xinjiang 830000, China; ^3^School of Chemical Engineering and Technology, Tianjin University, Tianjin 300350, China; ^4^Beijing Key Laboratory of Traditional Chinese Medicine Protection and Utilization, Faculty of Geographical Science, Beijing Normal University, Beijing, China; ^5^Analytic and Testing Center, Beijing Normal University, Beijing 100875, China

## Abstract

A nuclear magnetic resonance- (NMR-) based metabolomics method was used to identify differential metabolites of methanol extracts obtained from six parts of *Peganum harmala* L. (*P. harmala*), namely, the root, stem, leaf, flower, testa, and seed. Two multivariate statistical analysis methods, principal component analysis (PCA) and partial least squares-discriminant analysis (PLS-DA), were combined to clearly distinguish among the *P. harmala* samples from the six different parts. Eleven differential components were screened by the PLS-DA loading plot, and the relative contents were calculated by univariate analysis of variance. Chemometric results showed significant differences in the metabolites of the different parts of *P. harmala*. The seeds contained large amounts of harmaline, harmine, and vasicine compared to other organs. The acetic acid, proline, lysine, and sucrose contents of the roots were significantly higher than those of the other parts. In the testa, the vasicine, asparagine, choline, and 4-hydroxyisoleucine contents were clearly dominant. The obtained data revealed the distribution characteristics of the metabolomes of the different *P. harmala* parts and provided fundamental knowledge for the rational development of its medicinal parts.

## 1. Introduction

*P. harmala* is the only salt-tolerant perennial herb in the *Peganum* genus of the family Zygophyllaceae [1]. It has been used as a popular traditional ethnodrug for a long time due to its phytotherapeutic value [2]. Traditionally, the seeds have been used to relieve pain, to promote blood circulation, and to treat rheumatism and illnesses such as cough and asthma [3, 4]. The whole plant has been used for pain relief and as a rheumatism treatment [5]. *P. harmala* extract is a rich source of bioactive substances [6], including large amounts of primary and secondary metabolites [7]. The whole plant and its seeds contain several kinds of alkaloids [8]. The alkaloid content of the seeds accounts for 3.92–7% of the dry weight [9]. The main alkaloid components are derivatives of quinazoline and *β*-carboline [10], which have exhibited anticancer and antibacterial activities in pharmacological studies [4, 10]. Because these metabolites located in different parts of the plant can vary greatly in type and quantity, the resulting pharmacological activities and antibacterial effects are significantly different [11]. Previous studies mainly focused on the extraction and activities of alkaloid compounds in *P. harmala* [12]. Only few alkaloids (*β*-carbolines and quinazoline derivatives) isolated from the different organs in *P. harmala* have been investigated [4, 13, 14], and no comprehensive studies of the metabolome of each of its parts have been performed.

Plant metabolomics is a method for investigating the dynamic changes in small-molecule metabolites or components in plants, and it has played an increasingly important role in explaining plant growth and reproduction [15, 16]. However, it is challenging to use metabolomics to comprehensively annotate metabolites and analyse the physiological and ecological roles of metabolomes [17]. As a rule, the structure of a compound is determined by spectral methods after being isolated and purified by various chromatographic techniques [13]. This process is time-consuming and laborious. At present, nuclear magnetic resonance (NMR) metabolomics technology offers a fast and sensitive method to detect distinctive signals and resolve the structures of compounds in a mixture by comparing with available data [18]. This technique has been widely applied in pharmacology, pharmacodynamics, and pharmacokinetics studies because of its advantages, including simple preparation, its unbiased nature, and its ability to qualitatively and quantitatively detect multiple metabolic components simultaneously [19].

In the current study, an NMR-based plant metabolomics method was used to analyse fingerprint spectra of methanol extracts obtained from different parts of *P. harmala*. A multivariate unsupervised analysis method, namely, principal component analysis (PCA), was employed to distinguish between the metabolomes of six different *P. harmala* parts. To enlarge the difference found in the PCA model and detect influential variables, partial least squares-discriminant analysis (PLS-DA), a supervised pattern recognition method, was used to recognize the characteristic differential metabolites among the groups classified according to different organs of plants. The relative contents of the major metabolites were analysed to help in explaining their ecological significance. This investigation of the characteristic components of the metabolomes of different *P. harmala* organs provides a molecular-level understanding of the distribution pattern of the metabolomes in this plant, which is expected to facilitate the rational development of medicinal plant resources.

## 2. Materials and Methods

NaH_2_PO_4_ and Na_2_HPO_4_ were used to prepare the buffer (pH = 6). Methanol and NaN_3_ were used to inhibit the activity of the decomposing enzymes in the samples (analytical pure, Beijing Chemical Plant). A 1 mmol/L trimethylsilylpropanoic acid (TSP) solution was prepared using D_2_O (99.9% deuterated, Cambridge Isotope Laboratories, USA). The deuterated 3-(trimethylsilyl)propionate sodium (TSP, 99% purity, J&K Scientific Co., Ltd.) solution was used as the internal standard. Milli-Q deionized water was used in the experiments. The *P. harmala* plant samples were collected in the Changji prefecture of Xinjiang, China. The flowers, stems, roots, testas, seeds, and leaves of the samples were collected, dried in air, ground, dried to a constant weight, and stored in a desiccator until use.

Sample preparation: first, 100 mL of methanol was added to a precisely weighed 2.0000 g crushed and dried plant sample three times for ultrasonic extraction. After centrifugation for 10 minutes, the supernatants were combined and subjected to rotary evaporation. The sample was freeze-dried to obtain the extract. Then, 0.2 mL of the deuterated TSP solution (1 mmol), 0.3 mL of a phosphate buffer (pH = 6.0), and 0.2 mL of NaN_3_ (10 mmol) solution were added to 10 mg of the extract, which was precisely weighed and mixed well. After sonication and centrifugation, 0.6 mL of the supernatant was pipetted into a 5 mm tube for NMR analysis. Five parallel preparations were performed for each sample.

NMR experiments: ^1^H-NMR measurements were performed with a 500 MHz NMR spectrometer at 298K. ^1^H and ^13^C nuclear resonance frequencies were 500 and 125 MHz, respectively. For the one-dimensional hydrogen spectra, the noesygppr1d pulse sequence with suppressed water peaks was used with the following parameters: a water peak suppression power of 41 dB (Bruker nomenclature), pulse delay time of 2 s, scan number of 128, spectrum width of 12 ppm, pulse time of 9.8 *µ*s, sampling time of 2.72 s, relaxation time of 2.0 s, sampled data point number of 32,768, and free-induction decay (FID) resolution of 0.18 Hz. The FID was processed with an exponential window function with a widening factor of 0.3 Hz. The baseline adjustment and phase correction were all performed manually. The methanol-extracted, water-soluble metabolites were determined by NMR using the presaturation technique to suppress the water peaks; TSP was used as the internal standard, and deuterated water was used to lock the field. The ^1^H-NMR spectra were heavily superimposed, and it was difficult to identify many of the signals. Two-dimensional nuclear magnetic resonance experiments, including correlated spectroscopy (COSY), heteronuclear single quantum coherence (HSQC) spectroscopy, and heteronuclear multiple bond correlation (HMBC) spectroscopy, enabled the facile identification of the metabolites from the collected signals of the methanol extracts of the different *P. harmala* plant parts. For the COSY spectra, the spectral width was 5000 Hz, the number of sampling points was 400 (F1) × 4096 (F2), and the number of time increments was 24. For the HSQC spectra, the ^1^H and ^13^C spectral widths were 5000 Hz and 22638 Hz, respectively; the number of sampling points was 256 (F1) × 2048 (F2), and the number of time increments was 60. For the HMBC spectra, the ^1^H and ^13^C spectral widths were 5000 Hz and 30184 Hz, respectively; the number of sampling points was 256 (F1) × 4096 (F2), and the number of time increments was 112.

NMR data analysis: all the ^1^H-NMR spectra were processed using the TopSpin 3.2 software (Bruker Biospin). The baseline and phase were calibrated manually. After the chemical shifts were calibrated using TSP (*δ*_H_ = 0.00), the spectra were imported into the MestReNova software (version 8.0.1, Mestrelab Research, Santiago de Compostela, Spain) for data processing. After calibrating the phase and adjusting the baseline, the spectrum was integrated in the range of *δ*_H_ 0.5–10 ppm with an integration interval of 0.02 ppm. However, the spectrum was not integrated in the range of *δ*_H_ 4.71–5.05 ppm (residual water peak). The sum of the integral areas of the spectrum was normalized to generate an Excel data file, which was imported into MATLAB (Umetrics, Umea, Sweden). PCA and PLS-DA were performed after mean centre processing, and the differential metabolites were screened using a variable importance factor (VIP) of >1 in the loading model. The effectiveness of the PLS-DA model was validated by a permutation test. The relative contents of some metabolites were analysed by analysis of variance (ANOVA) and *t*-tests. Duncan's new multiple range test was used to correct the *p* value [20].

## 3. Results and Discussion

### 3.1. ^1^H-NMR Fingerprint Peak Assignment for the Methanol Extracts of the Different Parts of *P. harmala*

**Figure 1:**
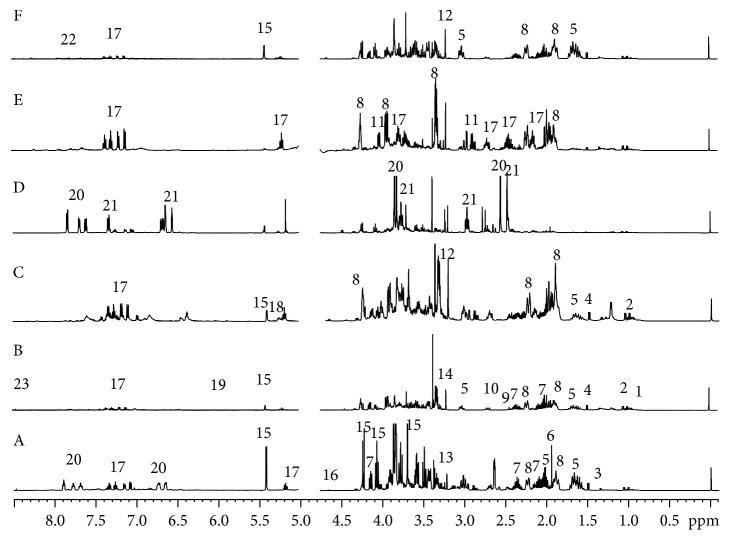
Typical 500 MHz ^1^H-NMR spectra of the metabolites of the different parts of *P. harmala*: (A) root, (B) leaf, (C) flower, (D) seed, (E) testa, and (F) stem. Key metabolites: 1, leucine; 2, valine; 3, threonine; 4, alanine; 5, lysine; 6, acetic acid; 7, proline; 8, 4-hydroxyisoleucine; 9, succinic acid; 10, malic acid; 11, asparagine; 12, choline; 13, phosphorylcholine; 14, betaine; 15, sucrose; 16, *β*-glucose; 17, vasicine; 18, α-glucose; 19, maleic acid; 20, harmine; 21, harmaline; 22, vasicinone; 23, formic acid.

The fingerprint spectra of the polar extracts of *P. harmala* revealed the presence of 23 metabolites, 19 primary and 4 secondary (Figure 1, Table 1). The 19 primary metabolites were three carbohydrate compounds, five organic acids, eight amino acids, and the last three were compounds of other types. The four secondary metabolites were vasicine, vasicinone, harmine, and harmaline. These twenty-three compounds were identified in the methanol extracts of *P. harmala* by comparing the chemical shifts and coupling splitting values in the nuclear magnetic spectra and the relevant information from the two-dimensional NMR spectra to related literature data [21] and the standard spectra of amino acids, organic acids, and sugar compounds in the Human Metabolome Database [22] (HMDB) (http://www.hmdb.ca/). The ^1^H-NMR spectra of methanol extracts from different organs of *P. harmala* and the characteristic signals of the identified metabolites are shown in Figure 1. The main amino acids identified in the organic acid and amino acid region (*δ*_H_ 0.5–3.0 ppm) were isoleucine, valine, threonine, alanine, proline, lysine, 4-hydroxyisoleucine, and asparagine. The main organic acids identified in this region included acetic acid, succinic acid, and malic acid. The signals of the sugar compounds in the range of *δ*_H_ 3.0–6.0 ppm overlapped considerably. However, diagnostic anomeric proton signal of sucrose and glucose could be easily identified. Meanwhile, the characteristic signals of nitrogen-containing metabolites, such as choline, phosphorylcholine, and betaine, were clearly observed in this region, as shown in Table 1.

In the aromatic region, vasicine, vasicinone, harmaline, and harmine were identified in the methanol extracts of *P. harmala*. Moreover, a comparison with the literature [23–25] and analysis of the two-dimensional COSY, HMBC, and HSQC spectra further confirmed the existence of these compounds, and the peak assignments are shown in Table 1. In addition to alkaloids described above, signal related to formic acid was also identified in the aromatic region of the spectra.

### 3.2. Multivariate Analysis of the ^1^H-NMR Data

**Figure 2:**
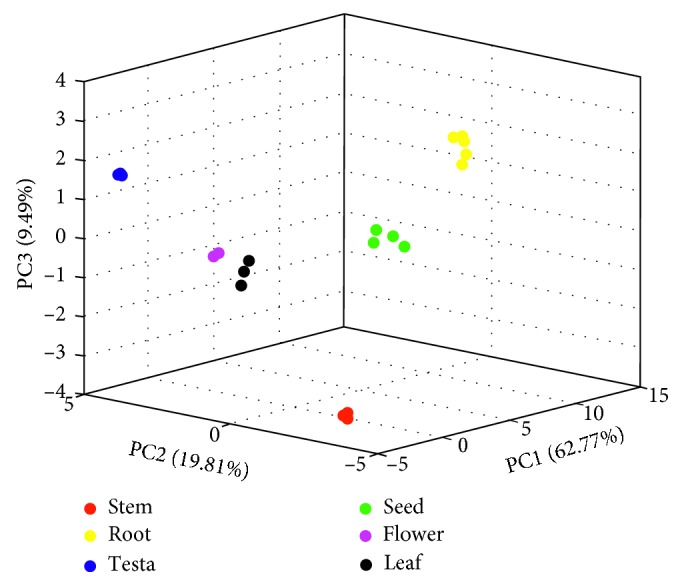
PCA score plot of the NMR spectra of the methanol extracts of the six different *P. harmala* parts (“red,” “yellow,” “blue,” “green,” “magenta,” and “black” dots represent the stem, root, testa, seed, flower, and leaf of the plant, respectively).

The ^1^H-NMR fingerprint spectra of the methanol extracts of the six parts of *P. harmala* are shown in Figure 1. A visual observation of the spectra revealed that the presence of many types of metabolites in stems, leaves, roots, and flowers, especially amino acids, with relatively high contents, whereas the metabolite levels in the testas and seeds were low. To further determine the potential differential metabolites, a multivariate statistical analysis (PCA) of the nuclear magnetic data of the polar metabolites of the six different *P. harmala* parts was performed, and the results are shown in Figure 2.

PCA is the most commonly performed unsupervised pattern recognition method in metabolomics research. The latent variable information can be determined from massive data sets to reduce the data dimensionality [26]. The sample classification information can be obtained from the score plot. In the current work, PCA was undertaken on the obtained data, and then satisfied results were generated. As shown in the PCA score plot in Figure 2, the first three principal components (PC1, PC2, and PC3) accounted for 92.07% of the original variable information (PC1: 62.77%, PC2: 19.81%, and PC3: 9.49%). The metabolites of the six *P. harmala* parts were significantly different, and the separation trend was obvious. Along the first ordination axis, the seeds and roots were clearly distinguished from the other plant parts and exhibited a positive correlation. In the PC2 direction of the score plot, the stems and roots were clearly distinguished from the extracts of the other plant parts, exhibiting a negative correlation.

**Figure 3:**
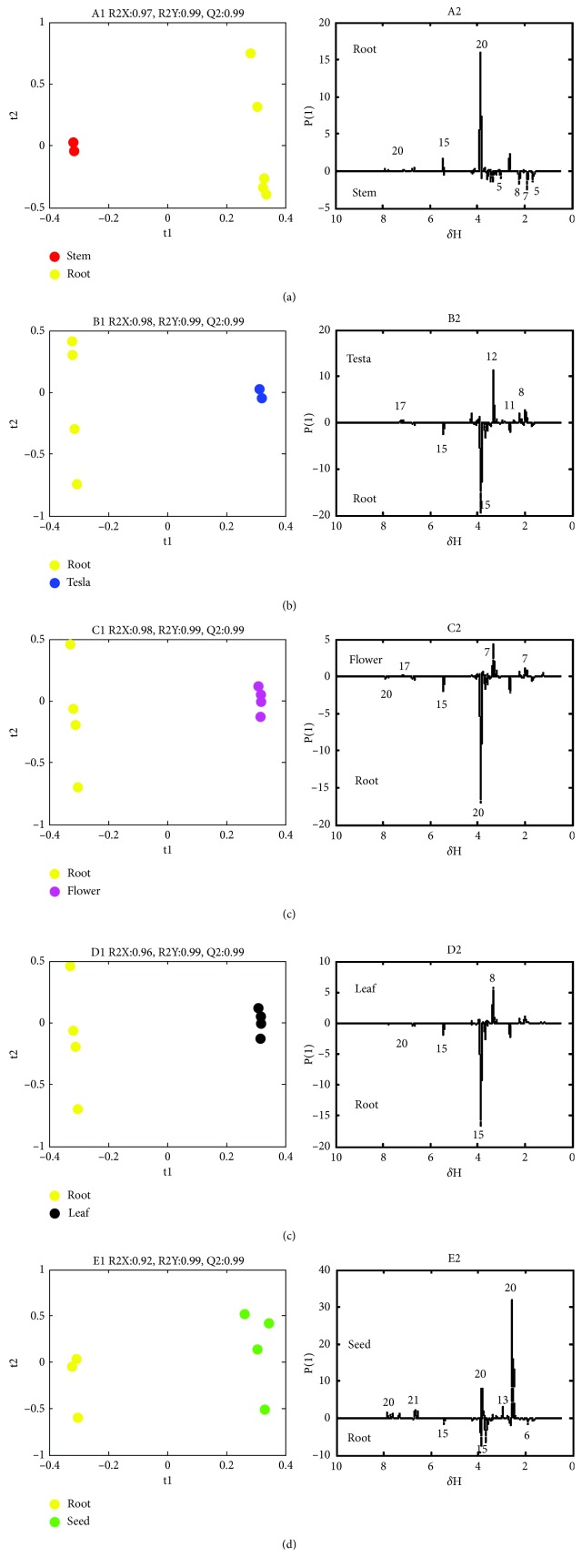
PLS-DA score plot (A1-E1) and linear loading plot (A2-E2) of the methanol extracts of the different *P. harmala* parts (“red,” “yellow,” “blue,” “green,” “magenta,” and “black” dots represent the methanol extracts of the stem, root, testa, seed, flower, and leaf of the plant, respectively; t1 and t2 are the scoring points for the first and second principal components, respectively; *δ*_H_ is the ^1^H-NMR chemical shift; P[1] is the loading point of the first principal component. The numbers represent different metabolites, as shown in Table 2).

To identify the differential metabolites of the six parts, the root sample was used as the control and the samples of the other parts were compared to it and sorted to identify the metabolites that contribute the most to the group classification. PLS-DA was applied to the ^1^H-NMR data of the stem, testa, flower, leaf, and seed to obtain score and loading plots (Figure 3). The score plot of A1, B1, C1, D1, and E1 in Figure 3 shows that the root was completely separated from the other parts. The parameters R2X, R2Y, and Q2 of the five models are shown in Figure 3 and were all greater than 0.9, indicating that the models had strong predictive power [27, 28]. Permutation tests were performed to PLS-DA models to verify that these parameters generated by the established models were not overfitted. Therefore, the models were validated, showing that the results were reliable. The loading plot can be used to determine the variables contributing to the classification, and depending on the levels of their contributions, the variables that can be considered as potential biomarkers can be discovered. Based on the A2, B2, C2, D2, and E2 groups shown in the PLS-DA loading plot, the significant differential metabolites included acetic acid (6), asparagine (11), lysine (5), proline (7), choline (12), phosphocholine (13), 4-hydroxyisoleucine (8), sucrose (15), vasicine (17), harmine (20), and harmaline (21).

### 3.3. Screening and Univariate Analysis of the Potential Characteristic Metabolites

The data with VIP>1 in the PLS-DA loading plot of the five methanol extracts of *P. harmala* were analysed to obtain the most significant metabolites for their classification, which included acetic acid, asparagine, choline, harmine, harmaline, 4-hydroxyisoleucine, phosphocholine, proline, lysine, sucrose, and vasicine. Specifically, to obtain the relative contents of the metabolites, which were subjected to univariate analysis of variance, their characteristic peaks were compared with the peak area of the internal standard. The calculated relative contents of the metabolites in the methanol extracts of the different *P. harmala* parts are shown in Table 2. All the data were analysed by ANOVA and *t*-tests. Differences were considered to be statistically significant at *p* < 0.05. The data in Table 2 show that the acetic acid, proline, lysine, sucrose, and vasicine contents of the *P. harmala* roots were significantly higher than those of the other parts. In addition to vasicine, the asparagine, choline, and 4-hydroxyisoleucine contents in the testas were clearly dominant. The seeds contained large amounts of harmaline, harmine, and phosphocholine. However, the differences in the metabolomes of the *P. harmala* stems, leaves, and flowers were not significant, which is consistent with the results of the multivariate analysis.

### 3.4. Biological Significance of the Characteristic Metabolites

The results showed that the sucrose, proline, acetic acid, betaine, and lysine contents of the *P. harmala* roots were much higher than those of the other parts. Of these metabolites, sucrose, proline, betaine, and acetic acid are osmotic regulators in plants, controlling osmotic adjustment [29]. The roots of a plant are the vegetative organ responsible for the physiological functions of nutrient absorption, production, and transport [30]. Sucrose is also used as an energy carrier in plants and is the main source of carbon and energy for plant growth and development. *P. harmala* roots are well suited to performing the main nutrient functions for plant growth and development and to contributing to plant metabolism, and sucrose provides energy for the growth, development, and reproduction of the plant [31]. The proline and betaine contents can increase under abiotic stress conditions, such as exposure to drought, high salinity, high temperatures, and heavy metals [32]. Besides to serve as valuable sources of nitrogen and carbon, proline and betaine prevent plant damage caused by osmotic stress and act as free radical scavengers [33–35]. It is generally believed that proline is mostly synthesized in the roots and most of the product is transported to the above-ground parts. The acidic substances in the roots can enhance the effectiveness of the nutrients in the rhizosphere soil, promote the acidification, chelation, ion exchange, and reduction in the insoluble nutrients in the plant, and participate in circulation and energy flow of the nutrient elements through the plant [36].

The experimental results showed that the alkaloid contents of the *P. harmala* seeds, including the harmine, harmaline, and phosphocholine contents, were most abundant compared to other parts [37]. Alkaloids are secondary metabolites that are generated when plants resist invasion from the environment and defend against external factors. They do not directly participate in plant growth, development, and reproduction, but they can improve the adaptability of the plant to adverse environments. The seed of *P. harmala* is the reproductive organ and is rich in alkaloids. To germinate and grow in an arid environment, the plant must strongly compete for water, nutrients, and space. The alkaloids in *P. harmala* seeds must be washed out by rainwater before germination. When these compounds enter the soil environment, they might inhibit the germination of other plant seeds and the growth of their seedlings through allelopathy [38]. The results demonstrate a higher concentration of phosphocholine in *P. harmala* seeds than other organs. The phosphate in the plant fluid exists mainly in the form of phosphocholine, a phosphorus carrier that accounts for 5–20% of the total phosphorus in plants, and is a key component of the plant cell membrane [39].

The data show an increase in the level of vasicine, asparagine, choline, and 4-hydroxyisoleucine contents in testa than the other groups. The testa is a protective layer on the outside of the plant seed that protects the seed embryo from mechanical damage and prevents pests, disease invasion, and water loss [40]. Its main physiological role is to control the germination of the seed by enhancing its dormancy, limit unfavourable biochemical activities during seed storage, and protect the seed and root from animal foraging and microbiological and viral invasion [41]. Harmaline and choline in the *P. harmala* testa might play a role in these defensive functions. 4-Hydroxyisoleucine is a unique nonprotein amino acid in plants that exhibits physiological and chemical activities that are responsible for glucose and lipid metabolism [42]. The amino acid asparagine is a soluble form of nitrogen that is predominantly absorbed directly by the plant roots. These compounds in the testa of *P. harmala* are closely related to its self-protection capabilities [42].

## 4. Conclusions

In this study, NMR was used to analyse methanol extracts of six parts of *P. harmala* and the plant metabolomics technique aimed at investigating the characteristic chemical composition of these extracts. The results showed that the differential metabolites in the different *P. harmala* parts included sucrose, harmine, proline, lysine, betaine, acetic acid, harmaline, vasicine, choline, 4-hydroxyisoleucine, and asparagine. From the quantitative point of view, the relative contents of these 11 metabolites in the methanol extracts of the six *P. harmala* parts studied were determined. The results revealed the significant differences in the types of metabolites in the *P. harmala* roots, seeds, and testas. The NMR-based plant metabolomics method established in this research can provide a detailed metabolomic profile of biological samples from different plant parts and allow the holistic analysis of metabolome. Resulting in improved understanding of *P. harmala* metabolism, the results can provide important data to aid in plant ecophysiological studies and facilitate the development of *P. harmala* medicinal resources.

## Figures and Tables

**Table 1:** ^1^H-NMR assignments of major metabolites in *P. harmala* extracts.

No.	Metabolite	*δ*_H_ (multiplicity, *J*)	Sample
1	Isoleucine	1.02 (d, 7.06), 0.95 (t, 7.15)	B^b^
2	Valine	1.05 (d, 7.0), 0.99 (d, 7.0)	B^b^
3	Threonine	4.25 (m), 1.33 (d, 6.55)	A^b^
4	Alanine	3.57 (m), 1.48 (d, 7.3)	B^b^
5	Lysine	3.6 (m), 1.65, 1.89 (m), 2.25 (m), 3.02, 3.4 (m)	F^b^
6	Acetic acid	1.92 (s)	A^b^
7	Proline	3.30–3.35 (m), 2.31–2.37 (m), 1.95–2.00 (m), 4.12 (dd, 8.63, 6.56)	A^b^
8	4-Hydroxyisoleucine	4.25 (m), 2.167 (m), 3.835 (m), 2.22, 1.98 (m), 1.88 (m)	E^a^
9	Succinic acid	2.43 (s), 2.43 (s)	F^b^
10	Malic acid	2.71 (dd, 2.9, 15.62), 4.31 (dd, 3.1, 10.2)	F^b^
11	Asparagine	4.03 (dd, 4.4, 7.25), 2.94 (m), 2.84 (m)	E^b^
12	Choline	4.07, 3.21 (s)	C^b^
13	Phosphorylcholine	3.23 (s)	A^b^
14	Betaine	3.27 (s)	B^b^
15	Sucrose	3.69 (s), 4.22 (d, 8.65), 4.06 (t, 8.89), 3.91 (m), 3.87 (d, 3.15), 5.42 (d, 3.8), 3.59 (m), 3.78 (t, 9.48), 3.49 (t, 9.28), 3.91(m)	A^b^
16	*β*-Glucose	4.65 (d, 8.0)	A^b^
17	Vasicine	3.77, 3.69 (m),2.15, 2.71 (m), 5.22 (t, 8.80), 7.11 (m), 7.36 (m), 7.28 (m), 7.19 (d, 7.5)	E^a^
18	*α*-Glucose	5.24 (d, 3.83)	C^b^
19	Maleic acid	6.01 (s)	B^b^
20	Harmine	6.64 (d, 1.9), 6.69 (dd, 8.75, 2.25), 7.84 (d, 5.64), 2.55 (s), 7.69 (d, 5.42), 7.61 (d, 8.59), 3.84 (s)	D^a^
21	Harmaline	6.46 (dd, 8.85, 1.94), 6.37 (d, 1.66), 7.0 (d, 8.64), 2.47 (s), 2.96(t, 8.83), 3.22(t, 8.83), 3.81(s)	D^a^
22	Vasicinone	4.11, 3.91 (m), 1.99, 2.45 (m), 4.99 (t, 6), 7.71 (m), 7.93 (m), 7.64 (m), 8.25 (d, 6)	F^a^
23	Formic acid	8.46 (s)	A^b^

*Note.* “a” was determined by the ^1^H-NMR, COSY, HSQC, and HMBC spectra. “b” was determined by the ^1^H-NMR and HSQC spectra. s, singlet; d, doublet; t, triplet; m, multiplet; A, root; B, leaf; C, flower; D, seed; E, testa; F, stem.

**Table 2:** Relative quantification of principal metabolites in different parts of *P harmala* (mg/g).

Compounds	Stem	Root	Testa	Seed	Flower	Leaf
Proline	37.88 ± 18.95bc	86.53 ± 10.27a	26.70 ± 2.14c	—	55.10 ± 7.88b	55.93 ± 2.39b
Lysine	153.63 ± 5.41b	180.02 ± 12.46a	—	—	95.87 ± 12.23c	68.85 ± 4.08d
Asparagine	17.77 ± 1.75c	17.96 ± 3.32c	123.84 ± 12.11a	29.37 ± 29.68c	76.03 ± 9.36b	32.03 ± 4.48c
4-Hydroxyisoleucine	204.85 ± 9.81b	189.29 ± 16.19b	364.89 ± 50.07a	—	195.70 ± 15.17b	160.35 ± 8.92b
Acetic acid	1.85 ± 0.17c	7.77 ± 0.98a	4.39 ± 0.85b	—	4.18 ± 0.54b	2.68 ± 0.09c
Sucrose	113.024 ± 8.45b	339.19 ± 35.69a	13.29 ± 4.92d	63.28 ± 2.0c	60.08 ± 5.91c	40.63 ± 1.63c
Vasicine	17.19 ± 9.8b	64.31 ± 8.44ab	124.13 ± 71.58a	—	68.36 ± 6.69ab	26.69 ± 2.56b
Harmine	—	19.27 ± 1.68b	—	40.77 ± 5.98a	—	—
Harmaline	—	—	—	58.46 ± 9.61	—	—
Choline	4.24 ± 0.17c	2.67 ± 0.18d	8.59 ± 0.96a	2.31 ± 1.45d	6.12 ± 0.51b	3.31 ± 0.19cd
Phosphorylcholine	0.55 ± 0.13d	2.92 ± 0.20b	3.56 ± 0.52b	5.50 ± 1.33a	0.97 ± 0.14cd	1.99 ± 0.13bc

*Note.* The numbers are the contents of the compounds in the methanol extracts of the different *P. harmala* parts (mean value ± standard deviation). “—” indicates that the compound was not detected. Duncan's new multiple range test was used to calculate the *p* value (when *p* < 0.05, different letters within same row represent subsets with significant differences; the order of the letters indicates the range of sample concentrations from large to small).

## Data Availability

The Excel data used to support the findings of this study are included within the supplementary information file.

## Supplementary Materials

Supplementary Materials Supplemental Table: the ^1^H-NMR raw data of the different parts of *P. harmala* methanol extract.
